# Effectiveness of personal letters to healthcare professionals in changing professional behaviours: a systematic review protocol

**DOI:** 10.1186/s13643-021-01650-4

**Published:** 2021-04-02

**Authors:** Aikaterini Grimani, Louis Goffe, Mei Yee Tang, Fiona Beyer, Falko F. Sniehotta, Ivo Vlaev

**Affiliations:** 1 NIHR Policy Research Unit in Behavioural Science, Newcastle University, Newcastle, UK; 2 Warwick Business School, University of Warwick, Coventry, UK; 3 Population Health Sciences Institute, Newcastle University, Newcastle, UK

**Keywords:** Healthcare professionals, Personal letters, Behavioural change, Systematic review

## Abstract

**Background:**

Letters are regularly sent by healthcare organisations to healthcare professionals to encourage them to take action, change practice or implement guidance. However, whether letters are an effective tool in delivering a change in healthcare professional behaviour is currently uncertain. In addition, there are currently no evidence-based guidelines to support health providers and authorities with advice on how to formulate the communication, what information and behaviour change techniques to include in order to optimise the potential effect on the behaviour of the receivers. To address this research gap, we seek to inform such guidance through this systematic review, which aims to provide comprehensive evidence of the effectiveness of personal letters to healthcare professionals in changing their professional behaviours.

**Methods/design:**

A comprehensive literature search of published and unpublished studies (the grey literature) in electronic databases will be conducted to identify randomised controlled trials (RCTs) that meet our inclusion criteria. We will include RCTs evaluating the effectiveness of personal letters to healthcare professionals in changing professional behaviours. The primary outcome will be behavioural change. The search will be conducted in five electronic databases (from their inception onwards): MEDLINE, Embase, PsycINFO, the Cochrane Library and CINAHL. We will also conduct supplementary searches in Google Scholar, hand search relevant journals, and conduct backward and forward citation searching for included studies and relevant reviews. A systematic approach to searching, screening, reviewing and data extraction will be applied in accordance with the process recommended by the Cochrane Collaboration. Two researchers will examine titles, abstracts, full-texts for eligibility independently. Risk of bias will be assessed using the Cochrane Risk of Bias 2 (RoB 2) tool for randomised controlled trials. Disagreements will be resolved by a consensus procedure.

**Discussion:**

Health policy makers across government are expected to benefit from being able to increase compliance in clinical settings by applying theories of behaviour to design of policy communications. The synthesised findings will be disseminated through peer-reviewed publication.

**Systematic review registration:**

PROSPERO CRD42020167674

**Supplementary Information:**

The online version contains supplementary material available at 10.1186/s13643-021-01650-4.

## Background

Letters are regularly sent by healthcare organisations to healthcare professionals (HCPs) to encourage them to take action, change practice or implement guidance (e.g. reduce prescribing of antibiotics [1] and sedative hypnotics [2], and raise performance of mental health clinicians [3]). Letters are inexpensive, usually personalised and have wide reach [1]. Letter is commonly defined as ‘a written, typed, or printed communication, sent in an envelope by post or messenger’ [4–6]. In addition, emails are usually defined as ‘written messages distributed by electronic means via a network’ [4].

Letters are promising in delivering behaviour change, especially when utilising particular behaviour change techniques (BCTs) boost the anticipated changes in behaviour. For example, Hallsworth [1] found that a letter containing a social norm message sent to general practitioners (GPs) notifying them that their practice is prescribing antibiotics at a higher rate than 80% of the practices in their local area reduced the prescribing rate compared to GPs who did not receive a letter. This study also revealed that a message from a high-profile sender (known as messenger effect) substantially reduced antibiotic prescribing at low cost and at national scale.

Whether letters are an effective tool in delivering a change in HCP behaviour is currently uncertain. The content of an intervention can be described through the use of BCTs which can be helpful in showing which particular techniques may contribute to intervention effectiveness. BCTs are the discrete and definable components of the given interventions that on their own have the potential to change behaviour. BCTs are observable and replicable and can be used alone or in combination with other BCTs [7]. A BCT is an ‘active ingredient’ such as *self-monitoring of behaviour* (e.g. keeping a daily diary of food consumption) [8]. Effective BCTs have been identified for interventions to increase physical activity and healthy eating and to support smoking cessation, safe drinking, prevention of sexually transmitted infections and changing professional behaviour [7]. However, there is currently no evidence about which BCTs [7] are used in letters, and how they might contribute to behaviour change.

Other approaches such as the MINDSPACE framework [9] could also identify and optimise the effects of letters to promote behaviour change. The MINDSPACE framework consists of nine evidence-based principles of behaviour change—messenger, incentives, norms, defaults, salience, priming, affect, commitment and ego—is becoming widely used within the policymaking community to improve the effectiveness of existing and new behaviour change policies [9, 10]. Likewise, there are a range of approaches to enhance behaviour change responses to letters, such as adding additional materials (e.g. a leaflet) or follow-up phone calls or text messages which might help boosting effects [1].

Despite the impact shown by selective studies for letters to be an effective tool in changing HCPs’ behaviours, to date, there has not been a comprehensive review to assess their potential in the context of population health. To address this research gap, we will conduct a systematic review that will identify what information and behaviour change techniques within letters that have the potential to optimise the effect on the behaviour of healthcare professionals.

## Objective

The aim of this research is to conduct a systematic review of RCTs evaluating interventions that include letters sent to HCPs to alter their professional behaviour and to identify lessons for improved letter design leading to behavioural compliance.

Research questions:

1. Are personal letters sent to HCPs from healthcare organisations effective in changing the clinical (e.g. immunisation, blood pressure measurement, prescription, referral) and non-clinical (e.g. achieve recruitment targets for clinical trials) behaviour of HCPs?
2. What are the BCTs within letters sent to HCP that have been evaluated?
3. What BCTs are effective in changing healthcare professional behaviour?
4. Does the effectiveness of letters sent to change healthcare professional behaviour vary by:
  - Characteristics of the sender
  - Characteristics of the receiver
  - Characteristics of the target behaviour (e.g. prescription, referral and diagnostic test)
  - Mode of message email vs. letter
  - Additional content (e.g. added leaflet)
  - Additional communications (e.g. follow-up calls, tests, reminder letters)
  - Other letter characteristics that may not be classified according to the existing BCT taxonomies (e.g. adding signature) [7, 9]

## Methods/design

This protocol has been registered within PROSPERO (number CRD42020167674) [11] and is being reported in accordance with the reporting guidance provided in the Preferred Reporting Items for Systematic Reviews and Meta-Analyses Protocols (PRISMA-P) statement [12, 13] (see checklist in Additional file 1). The systematic review will be reported following the preferred reporting items for systematic reviews and meta-analyses (PRISMA) statement [14, 15].

A comprehensive literature search will be conducted in five electronic databases (from inception onwards): Medline, EMBASE, PsycINFO, Cochrane Library and CINAHL. We will conduct supplementary searches in Google Scholar, hand search relevant journals, and conduct backward and forward citation searching of included studies and relevant reviews. To identify additional grey literature not (yet) published in peer reviewed journals, we will contact partners (e.g. UK government departments such as Department of Health and Social Care (DHSC); agencies and public bodies such as Public Health England; behavioural science units such as the Behavioural Insights Team). The titles and abstracts retrieved by electronic searching will be downloaded to the reference management database EndNote.

A search strategy (see draft search in Additional file 2) following PICOS (population, intervention, comparison, outcome and study design) will be written for one database and then translated, using appropriate syntax and Medical Subject Headings (MeSH) terms for other databases [16, 17]. The PICOS process facilitates a more evidence-based approach to literature searching and helps to rapidly and accurately locate the best available scientific information, avoiding unnecessary searching [17].

### Eligibility criteria

Published and unpublished (grey literature) studies written in English language (up to February 2021) will be considered. The specific inclusion criteria of the study will be the following:

▶ Population: We will include HCPs who are involved in providing direct patient care (e.g. mental health clinicians, primary care physicians, general practitioners; this includes HCPs in training). No restrictions will be placed on level of care (e.g. primary/secondary care) or geographical region.

▶ Interventions: We will be looking for letters sent from healthcare organisations addressed directly to HCPs with the intent to alter their professional practice. The personal letters could be either physical or electronic, and we will classify them separately in our analysis. For example, we will include letters written on paper or hard copy letters sent by postal service. We will also include interventions in which the letter is the content of an email or an attachment to an email.

▶ Comparisons: Relevant comparisons will include either no intervention control arms, or written information in any format (e.g., non-personalised information such as circulars or newsletters, letters with additional materials (such as leaflets) or reminders (by phone, text or letter)) sent to individual healthcare practitioners.

▶ Primary outcomes: We are interested in changes of HCPs’ behaviour that are due to the letters sent by healthcare organisations. In particular, we will include measures of HCP clinical behaviour such as rates of performing prevention (e.g. immunisation), diagnosis and treatment behaviours (e.g. blood pressure measurement, prescription, referral); and measures of HCP non-clinical behaviour such as rates of performing specified non-clinical behaviours (for example achieve recruitment targets for clinical trials). No secondary outcomes will be included.

▶ Types of studies to be included: We will include only RCTs. RCTs are considered the gold standard of study design to establish intervention effectiveness. We will include formats such as trials registries, conference papers, preprints, if sufficient information is available on study design, characteristics of participants, interventions and outcomes.

### Patient and public involvement (PPI)

PPI involvement has the ability to empower people with respect to health and social care services, influencing change and improvement in issues regarding these services and facilitating people to use them [18]. A core component of Policy Research Unit (PRU) policy is to maximise public involvement in all our research activities [19]. Thus, the PRU PPI panel has commented on the protocol and will receive regular updates on the review and comment on outputs.

### Selection of studies

Two review authors will independently examine the titles and abstracts retrieved by electronic searching after duplicates have been removed. The titles and abstracts will be included if they meet the inclusion criteria. Subsequently, two reviewers will independently determine the eligibility of studies on the basis of a review of the full texts. Differences in judgement will be resolved through discussion and inclusion of a third rater. The interrater reliability will be tested using Cohen’s kappa coefficient. Kappa result will be interpreted as follows: values ≤ 0 as indicating no agreement and 0.01–0.20 as none to slight, 0.21–0.40 as fair, 0.41–0.60 as moderate, 0.61–0.80 as substantial and 0.81–1.00 as almost perfect agreement. The percent agreement will also be calculated. Considering the strengths and limitations of these measurements, McHugh [20] has suggested the calculation of both percent agreement and kappa.

The elements of the systematic review will be conducted via Covidence, one of Cochrane’s recommended tools. This web-based software platform has been designed to support more efficient management of systematic reviews and can be used from the beginning of title/abstract screening through the beginning of meta-analysis [21]. The selection process will be recorded and the PRISMA flow diagram will be completed [12].

### Data extraction

The process of data extraction will involve two reviewers who will independently extract the data using a standardised data extraction form (see draft form in Additional file 3). The data extraction form, which will be piloted on a few studies, refined accordingly by the reviewers and finalised, will include the following variables:

▶ Characteristics of the receiver

▶ Characteristics of the sender

▶ Intervention characteristics including

- Template for Intervention Description and Replication (TIDieR) checklist: (brief name, why, what (materials), what (procedure), who provided, how, where, when and how much, tailoring, modifications, how well (planned), how well (actual) [22].
- MINDSPACE framework helps reframe the decision so that different contextual forces can be employed to induce behavioural changes: the nine most robust effects on behaviour (messenger, incentives, norms, defaults, salience, priming, affect, commitment and ego) [9, 23].
- BCTs (observable and replicable components designed to change behaviour) [7, 24], intervention functions (education, persuasion, incentivisation, coercion, training, restriction, environmental restructuring, modelling, enablement) and policy categories (communication/marketing, guidelines, fiscal, regulation, legislation, environmental/social planning, service provision), which are all part of the Behaviour Change Wheel framework [7, 25].
- Primary outcome(s): Measures of HCPs’ clinical and non-clinical behaviour. Where more than one reported outcome is provided, we will use the grading of recommendation, assessment, development and evaluation (GRADE) approach to assess the certainty of evidence (separated into those that are critical, important and not important) [26]. Outcomes measured at multiple time points will be categorised as following: immediate (within 2 weeks of the intervention delivery), short-term (2–13 weeks after intervention delivery), medium-term (14–50 weeks after intervention delivery) and long-term effects (51 or more weeks after intervention delivery). We will present multiple time points only for critical outcomes (changes in HCPs’ clinical and non-clinical behaviour).

### Quality assessment

An assessment of the methodological quality of included studies will be conducted using the Cochrane RoB 2 tool [27]. RoB 2 is the revised risk-of-bias assessment tool which provides a framework for considering the risk of bias in the findings of any type of randomised trial. It is structured into the following five bias domains: bias arising from the randomisation process; bias due to deviations from intended interventions; bias due to missing outcome data; bias in measurement of the outcome; bias in selection of the reported result. The response options are ‘yes’, ‘probably yes’, ‘probably no’, ‘no’ and ‘no information’. The risk-of-bias judgements for each domain are ‘low risk of bias’, ‘some concerns’ or ‘high risk of bias’. The studies, whose all domains will be rated low risk, will be judged as low risk of bias, whilst the studies with one or more concerns will be judged to raise some concerns. Furthermore, the studies, where one or more domains will be rated high risk, will be judged to be at high risk of bias [28]. Two review authors will independently evaluate the methodological quality of each included study using the assessment tool. Discrepancies will be resolved through a consensus procedure.

### Data analysis and synthesis

We will compare the reported *intervention characteristics* against a control condition, which does not contain those characteristics. This will provide information useful for explaining why interventions were effective or ineffective. The analyses of those comparisons will be conducted and reported separately according to the *HCPs’ characteristics* and *characteristics of the sender*.

BCTs will be double-coded by trained coders using the Behaviour Change Techniques Taxonomy v1 [7]. BCTs will be coded separately for intervention and control groups. The interrater reliability of coding of BCTs (namely the presence or absence of techniques within each intervention), reporting TIDieR and completing MINDSPACE will be assessed using the prevalence and bias-adjusted kappa (PABAK) statistic [29]. PABAK was used because it adjusts for shared bias in the coders’ use of categories and high prevalence of negative agreement (i.e. when both coders agree that codes are absent). A meta-regression analysis with the extracted BCTs will be conducted, if the data permits. The meta-analysis will be conducted when a group of studies are sufficiently homogeneous in terms of subjects involved, interventions and outcomes to provide a meaningful summary. Although a meta-analysis can be conducted with a minimum of two studies, Valentine, Pigott [30] suggest that the combination of very few studies with heterogeneous characteristics makes any kind of synthesis untenable in most cases, whilst parameter estimation (e.g. the random effects variance component) will likely be poor, rendering conclusions that are highly uncertain.

## Discussion

This knowledge synthesis can serve as a guide for effective interventions to support providers and health authorities with advice on how to formulate the communication, what information and BCTs to include in order to optimise the potential effect of letters on the behaviour of the receivers (HCPs). Drawing on the findings, we will develop a guide to help staff optimise communications with HCPs. Reaching people with a message is one thing, influencing and changing their behaviour is another. Our behavioural science review will provide tried and tested methods that help us to design communications that more effectively influence the decisions HCPs make. This guide will include a checklist that details step by step instructions on how to design, develop and test behaviourally informed communications. It will include tips, techniques and examples of how the methods outlined have been used successfully around the globe in recent years. We will try to keep the guide as simple and accessible as possible. It is expected to help quickly learn about the theory, steps and techniques that can be applied to design more effective communications. Whether we want our recipients to start, stop or change a behaviour, following the checklist will help influencers to get the response they want. In particular, the review is hoped to inform the development of the multi-step checklist, which will include sections such as Receiver, Action, Sender, Channel, Techniques. In particular:

▶ Receiver: Who is the target audience? This step includes also finding opportunities for segmenting the target audience—i.e. grouping HCPs together by tendencies and characteristics so one can tailor the message to suit the characteristics of each different group.

▶ Action: What is the desired response? This step involves clearly defining the behavioural response required by the recipient(s) of the communication.

▶ Sender: Who is the sender of the letter? The receiver may respond differently depending on who is the messenger (e.g. central government, local authority, senior manager, regulator, clinical/professional body, nongovernmental organisation, and private company).

▶ Channel: What is the best mode of letter delivery (e.g. post, email, sms, in person) to maximise engagement for the selected Recipient and Effect?

▶ Techniques: Which BCTs can be used to design the communications for the selected Recipient, Effect, and Channel? Whilst techniques come in many different forms, we will list the ones that have proven to be particularly effective and useful when designing communications. For example, people are more likely to respond when they feel they are being addressed as individuals and not just a ‘number’. Also, does the letter grab and hold the reader’s attention? Is the language simple, clear and easy to understand? Is there a clear call to action (i.e. can the reader understand what is being asked of them and by when within the first few lines of the document)? Does it use the right tone of voice?

The behavioural analysis, which will be carried out using the Behaviour Change Wheel framework, will identify the key behaviours and, importantly, drivers for behaviours that may be amenable to change. The review will inform the development of a multi-step checklist, which will include sections such as Receiver, Action, Sender, Channel, Techniques, giving important insights and practical applications for policymakers, health insurers, and health systems. The inclusion of grey literature will broaden this study in terms of included information. Limitations of this review include the exclusion of papers reported in languages other than English. However, as the abstracts of these papers are usually written in English, screening will be implemented and a list of the references seemed to fulfil the inclusion criteria will be provided in appendix.

We will base our conclusions on findings from the narrative synthesis of included studies for this review. Health policy makers across government will benefit from being able to increase compliance in clinical settings by applying theories of behaviour to the design of policy communications, especially considering that letters are commonly used by health systems to influence HCPs.

There is growing evidence that BCTs can positively influence behaviours in a range of clinical situations. However, it remains unclear what BCTs have been most effective and how physicians experienced intervention letters (e.g. as helpful feedback or as a scare tactic). Ultimately, this kind of multicomponent intervention may have been the most effective approach considering that the low cost of the letter means that even a relatively small effect size could be cost-effective. These are important insights for policymakers, health insurers and health systems as they jointly pursue efforts to improve the value of care.

## Supplementary Information

**Additional file 1:.** PRISMA-P 2015 Checklist

**Additional file 2: Table 1**. Sample search strategy terms (Medline)

**Additional file 3: Table 1**. General characteristics. **Table 2**. TIDieR. **Table 3**. MINDSPACE Framework for behaviour change. **Table 4**. BCTs (per study)

## Data Availability

Not applicable.

## Abbreviations

HCPs: Healthcare professionals
BCTs: Behavioural change techniques
GPs: General practitioners
PPI: Patient and public involvement
TIDieR: Template for intervention description and replication
GRADE: Grading of recommendation, assessment, development and evaluation
PABAK: Prevalence and bias-adjusted kappa
PRISMA: Preferred reporting items for systematic reviews and meta-analyses
Embase: Excerpta medica database
MEDLINE: Medical literature analysis and retrieval system online
CINAHL: Cumulative index to nursing and allied health literature
PICOS: Population, intervention, comparison, outcome, study design
RCT: Randomised control trial
MeSH: Medical subject headings
RoB 2 tool: Risk of bias 2 tool

## References

[1] Hallsworth M. Increasing compliance in policy settings by applying psychological theories of behaviour to message design. 2017.

[2] Smith DH, Christensen DB, Stergachis A, Holmes G (1998). A randomized controlled trial of a drug use review intervention for sedative hypnotic medications. Med Care.

[3] Azocar F, Cuffel B, Goldman W, McCarter L (2003). The impact of evidence-based guideline dissemination for the assessment and treatment of major depression in a managed behavioral health care organization. J Behav Health Serv Res.

[4] Stevenson A (2010). Oxford dictionary of English.

[5] McCreary DR (2009). Cambridge Academic Content Dictionary. Dictionaries J Dictionary Soc N Am.

[6] Staff M-W. Merriam-Webster’s collegiate dictionary: Merriam-Webster. Springfield: Massachusetts; 2004.

[7] Michie S, Richardson M, Johnston M, Abraham C, Francis J, Hardeman W, Eccles MP, Cane J, Wood CE (2013). The behavior change technique taxonomy (v1) of 93 hierarchically clustered techniques: building an international consensus for the reporting of behavior change interventions. Ann Behav Med.

[8] Cane J, Richardson M, Johnston M, Ladha R, Michie S (2015). From lists of behaviour change techniques (BCT s) to structured hierarchies: comparison of two methods of developing a hierarchy of BCT s. Br J Health Psychol.

[9] Dolan P, Hallsworth M, Halpern D, King D, Metcalfe R, Vlaev I (2012). Influencing behaviour: the mindspace way. J Econ Psychol.

[10] Yoong SL, Hall A, Stacey F, Grady A, Sutherland R, Wyse R, Anderson A, Nathan N, Wolfenden L (2020). Nudge strategies to improve healthcare providers’ implementation of evidence-based guidelines, policies and practices: a systematic review of trials included within Cochrane systematic reviews. Implementation Sci.

[11] Grimani A, Goffe L, Tang MY, Sniehotta F, Vlaev I. The effectiveness of personal letters to healthcare professionals in changing clinical practice behaviours: a systematic review: PROSPERO 2020 CRD42020167674; [Available from: https://www.crd.york.ac.uk/prospero/display_record.php?ID=CRD42020167674. 10.1186/s13643-021-01650-4 PMC8017654 33794987

[12] Moher D, Shamseer L, Clarke M, Ghersi D, Liberati A, Petticrew M (2015). Preferred reporting items for systematic review and meta-analysis protocols (PRISMA-P) 2015 statement. Syst Rev.

[13] Shamseer L, Moher D, Clarke M, Ghersi D, Liberati A, Petticrew M, et al. Preferred reporting items for systematic review and meta-analysis protocols (PRISMA-P) 2015: elaboration and explanation. Bmj. 2015;349(jan02 1). 10.1136/bmj.g7647. 10.1136/bmj.g7647 25555855

[14] Moher D, Liberati A, Tetzlaff J, Altman DG, Group P (2009). Preferred reporting items for systematic reviews and meta-analyses: the PRISMA statement. J Clin Epidemiol.

[15] Liberati A, Altman DG, Tetzlaff J, Mulrow C, Gotzsche PC, Ioannidis JP (2009). The PRISMA statement for reporting systematic reviews and meta-analyses of studies that evaluate health care interventions: explanation and elaboration. J Clin Epidemiol.

[16] Huang M, Névéol A, Lu Z (2011). Recommending MeSH terms for annotating biomedical articles. J Am Med Inform Assoc.

[17] Methley AM, Campbell S, Chew-Graham C, McNally R, Cheraghi-Sohi S (2014). PICO, PICOS and SPIDER: a comparison study of specificity and sensitivity in three search tools for qualitative systematic reviews. BMC Health Serv Res.

[18] Hayes H, Buckland S, Tarpey M (2012). Briefing notes for researchers: public involvement in NHS, public health and social care research.

[19] PRU Behavioural Science. Partnership working: patient and public engagement 2020 [Available from: https://research.ncl.ac.uk/behscipru/partnership%20working/.

[20] McHugh ML (2012). Interrater reliability: the kappa statistic. Biochem Med (Zagreb).

[21] Covidence systematic review software. Melbourne: Veritas Health Innovation; Available at www.covidence.org.

[22] Hoffmann TC, Glasziou PP, Boutron I, Milne R, Perera R, Moher D, et al. Better reporting of interventions: template for intervention description and replication (TIDieR) checklist and guide. Bmj. 2014;348(mar07 3). 10.1136/bmj.g1687. 10.1136/bmj.g1687 24609605

[23] Vlaev I, King D, Dolan P, Darzi A (2016). The theory and practice of “nudging”: changing health behaviors. Public Adm Rev.

[24] Michie S, Wood CE, Johnston M, Abraham C, Francis J, Hardeman W. Behaviour change techniques: the development and evaluation of a taxonomic method for reporting and describing behaviour change interventions (a suite of five studies involving consensus methods, randomised controlled trials and analysis of qualitative data). Health Technol Assess. 2015;19(99):1–188. 10.3310/hta19990 PMC4781650 26616119

[25] Michie S, Van Stralen MM, West R (2011). The behaviour change wheel: a new method for characterising and designing behaviour change interventions. Implementation Sci.

[26] Schünemann HJ, Higgins JP, Vist GE, Glasziou P, Akl EA, Skoetz N, et al. Completing ‘Summary of findings’ tables and grading the certainty of the evidence. In: Higgins JPT, Thomas J, Chandler J, Cumpston M, Li T, Page MJ, Welch VA (editors). Cochrane Handbook for Systematic Reviews of Interventions. 2nd Edition. Chichester: Wiley; 2019:375–402. 10.1002/9781119536604.ch14.

[27] Sterne JA, Savović J, Page MJ, Elbers RG, Blencowe NS, Boutron I, et al. RoB 2: a revised tool for assessing risk of bias in randomised trials. BMJ. 2019;366. 10.1136/bmj.l4898 31462531

[28] Higgins JP, Thomas J, Chandler J, Cumpston M, Li T, Page MJ (2019). Cochrane handbook for systematic reviews of interventions.

[29] Michie S, Wood CE, Johnston M, Abraham C, Francis JJ, Hardeman W. Training to code intervention descriptions using Behaviour Change Technique Taxonomy version 1 (study 3). Behaviour change techniques: the development and evaluation of a taxonomic method for reporting and describing behaviour change interventions (a suite of five studies involving consensus methods, randomised controlled trials and analysis of qualitative data): NIHR Journals Library; 2015. 10.3310/hta19990 PMC4781650 26616119

[30] Valentine JC, Pigott TD, Rothstein HR (2010). How many studies do you need? A primer on statistical power for meta-analysis. J Educ Behav Stat.

