# Supplementary material for: Effectiveness of personal letters to healthcare professionals in changing professional behaviours: a systematic review protocol
Source: Syst Rev. 2021 Apr 2;10:94. doi: 10.1186/s13643-021-01650-4 (PMC8017654; doi:10.1186/s13643-021-01650-4)
Supplement: Supplementary file 2 — Additional file 2: Table 1. Sample search strategy terms (Medline) [file 13643_2021_1650_MOESM2_ESM.docx]

**Table 1. Sample search strategy terms (Medline)**

| 1. (anaesthetist* or anesthetist* or audiologist* or cardiologist* or chiropodist* or clinician* or consultant* or cadet* or counsellor* or dentist* or dermatologist* or dietician* or Doctor* or GP or gynaecologist* or gynecologist* or matron* or midwife or midwives or neurologist* or nurse* or nutritionist* or obstetrician* or oncologist* or optometrist* or orthodontist* or orthoptist* or orthotist* or osteopath* or paediatrician* or pediatrician* or paramedic* or pathologist* or pharmacist* or phlebotomist* or physician* or physiologist* or physiotherapist* or podiatrist* or practice manager* or practice staff or practitioner* or prosthetist* or psychiatrist* or psychologist* or psychotherapist* or radiographer* or radiologist* or registrar* or rheumatologist* or surgeon* or therapist* or urologist* or anesthesiologist* or prescriber* or sonographer*).ab,ti. |
| --- |
| 1. ((ambulance or associate or audiology or cardiology or chiropody or clinical or dental or dermatology or family or gynaecologist* or gynecology or health or healthcare or "health care" or hospital or house or medical or midwifery or neurology or nursing or nutrition or obstetrics or oncology or optometry or orthodontic or paediatric* or pediatric* or pathology or pharmacy or physiology or physiotherapy or podiatry or psychiatry or psychology or "public health" or radiolog* or rheumatology or surgical or therapy or trainee or urology or respiratory or magnetic resonance imaging) adj2 (assistant* or cadet* or director* or manager* or officer* or personnel or practice or practitioner* or professional* or provider or receptionist* or resident* or scientist* or secretar* or specialist* or staff or technician* or technologist or visitor* or worker*)).ab,ti. |
| 1. 1 or 2 |
| 1. (letter* or post or posted or mail* or print communicat* or leaflet*).ab,ti. |
| 1. exp Correspondence As Topic/ |
| 1. ((electronic or web or online or internet) adj2 (mail* or messag* or communicat* or letter* or remind*)).ab,ti. |
| 1. ("e-mail" or "email").ab,ti. |
| 1. exp Electronic Mail/ |
| 1. 4 or 5 or 6 or 7 or 8 |
| 1. ((behaviour* or behavio?r*) adj5 (change or influenc* or alter* or modif*)).ab,ti. |
| 1. ((social or descriptive or peer or subjective) adj2 (norm or norms or comparison)).ab,ti. |
| 1. ((restructur* or add* or reduce or change or alter*) adj2 (social or physical or environment*)).ab,ti. |
| 1. 10 or 11 or 12 |
| 1. 3 and 9 and 13 |
| 1. exp Animals/ |
| 1. human/ |
| 1. 15 not (15 and 16) |
| 1. 14 not 17 |
