# Supplementary material for: Effectiveness of personal letters to healthcare professionals in changing professional behaviours: a systematic review protocol
Source: Syst Rev. 2021 Apr 2;10:94. doi: 10.1186/s13643-021-01650-4 (PMC8017654; doi:10.1186/s13643-021-01650-4)
Supplement: Supplementary file 3 — Additional file 3: Table 1. General characteristics. Table 2. TIDieR. Table 3. MINDSPACE Framework for behaviour change. Table 4. BCTs (per study) [file 13643_2021_1650_MOESM3_ESM.docx]

**Example Data Extraction Form**

Table 1. General characteristics

| Author, year of publication, title of study | Country | Design  (RCT; Case-control study; Cluster randomised controlled trial; controlled interrupted time series; historically controlled trial; prospective or retrospective cohort study) | Characteristics of the receiver(s) (number of HCPs, age, gender, residency etc.) | Characteristics of the sender (healthcare organisation etc.) | Intervention aimed to increase, decrease, or to maintain the target behaviour? | Type of control group | Total number of participants randomised (in total; for each intervention group) | Attrition (withdrawals/ exclusions before and after) | Measurement of the outcome(s) (objective or subjective) | Outcome(s) (HCPs' clinical and/ or non-clinical behaviours) |
| --- | --- | --- | --- | --- | --- | --- | --- | --- | --- | --- |
| Study 1 |  |  |  |  |  |  |  |  |  |  |
| Study 2 |  |  |  |  |  |  |  |  |  |  |
| Study n |  |  |  |  |  |  |  |  |  |  |

Table 2. TIDieR

|  | Brief name or a phrase that describes the intervention | Why  (any rationale, theory, or goal that underpin the intervention) | What materials  (any physical or informational materials used in the intervention) | What procedures (each of the procedures, activities, and/or processes used in the intervention) | Who provided (expertise, background and any specific training given for each category of intervention provider) | How  (modes of delivery of the intervention and whether it was provided individually or in a group) | Where  (type(s) of location(s) where the intervention occurred) | When and how much (number of times the intervention was delivered and over what period of time (number of sessions, their schedule, and their duration, intensity or dose)) | Tailoring  (If the intervention was planned to be personalised, titrated or adapted, (what, why, when, and how)) | "Modifications (If the intervention was modified during the course of the study, (what, why, when, and how)) | How well planned  (how and by whom, and if any strategies were used to maintain or improve fidelity; including the unit of allocation and type of RCT) | "How well actual  (the extent to which the intervention was delivered as planned) |
| --- | --- | --- | --- | --- | --- | --- | --- | --- | --- | --- | --- | --- |
| Study 1 |  |  |  |  |  |  |  |  |  |  |  |  |
| Study 2 |  |  |  |  |  |  |  |  |  |  |  |  |
| Study n |  |  |  |  |  |  |  |  |  |  |  |  |

Table 3. MINDSPACE Framework for behaviour change

|  | **Messenger**  (We are heavily influenced by who communicates with us)  *Yes/No (classifying influences below)* | **Incentives**  (Our responses to incentives are shaped by predictable mental shortcuts, such as strongly avoiding losses)  *Yes/No (classifying influences below)* | **Norms**  (We are strongly influenced by what other people do)  *Yes/No (classifying influences below)* | **Defaults**  (We tend to ‘go with the flow’ of pre-set options)  *Yes/No (classifying influences below)* | **Salience**  (Our attention is drawn to what is novel and seems relevant for us)  *Yes/No (classifying influences below)* | **Priming**  (Our actions are often influenced by sub-conscious clues)  *Yes/No (classifying influences below)* | **Affect**  (Our emotional associations can powerfully shape our actions)  *Yes/No (classifying influences below)* | **Commitments**  (We seek to be consistent with our public promises, and reciprocate acts)  *Yes/No (classifying influences below)* | **Ego**  (We act in ways that make us feel better about ourselves)  *Yes/No (classifying influences below)* |
| --- | --- | --- | --- | --- | --- | --- | --- | --- | --- |
| Study 1 |  |  |  |  |  |  |  |  |  |
| Study 2 |  |  |  |  |  |  |  |  |  |
| Study n |  |  |  |  |  |  |  |  |  |

Table 4. BCTs (per study)

| Author (year): |  | | | |
| --- | --- | --- | --- | --- |
| Title: |  | | | |
| Target population: |  | | | |
| Target behaviour: |  | | | |
| Arm (3) | | Coder 1 BCTs | Coder 2 BCTs | Final agreed BCTs |
| Control  *(Name)* | |  |  |  |
| *If guidelines present* | | Guideline unspecified (delete as appropriate): Yes/No (list BCTs below)  Guideline BCTs (list): | Guideline unspecified (delete as appropriate): Yes/No (list BCTs below)  Guideline BCTs (list): |  |
| Target Intervention  *(Name)* | |  |  |  |
| *If guidelines present* | | Guideline unspecified (delete as appropriate): Yes/No (list BCTs below)  Guideline BCTs (list): | Guideline unspecified (delete as appropriate): Yes/No (list BCTs below)  Guideline BCTs (list): |  |
| Additional Intervention  *(Name)* | |  |  |  |
| *If guidelines present* | | Guideline unspecified (delete as appropriate): Yes/No (list BCTs below)  Guideline BCTs (list): | Guideline unspecified (delete as appropriate): Yes/No (list BCTs below)  Guideline BCTs (list): |  |
